# ﻿A taxonomic review of the genus *Rhinoceros* with emphasis on the distinction of *Eurhinoceros* (Perissodactyla, Rhinocerotidae)

**DOI:** 10.3897/zookeys.1230.127858

**Published:** 2025-03-06

**Authors:** Francesco Nardelli, Kurt Heißig

**Affiliations:** 1 IUCN SSC Asian Rhino Specialist Group and Conservation Planning Specialist Group, Gland, Switzerland IUCN SSC Asian Rhino Specialist Group and Conservation Planning Specialist Group Gland Switzerland; 2 Bayerische Staatssammlung für Paläontologie und Geologie, München, Germany Bayerische Staatssammlung für Paläontologie und Geologie München Germany

**Keywords:** Ecology, *Eurhinocerossondaicus*, Indian rhinoceros, Javan, morphology, palaeontology, *Rhinocerosunicornis*, Sundaic rhinoceros, systematics

## Abstract

This study examines the ecomorphological characteristics of two Asian rhinoceros species: the critically endangered Sundaic rhinoceros and the vulnerable Indian rhinoceros. Among the five living rhinoceros taxa, the three Asian species are notable for their tusked incisors. Fossil evidence highlights the divergence between *Rhinoceros* and *Eurhinoceros* in cheek tooth morphology, linked to different dietary specialisations. The Sundaic rhinoceros, a generalist browser restricted to the Ujung Kulon peninsula of Java, exhibits distinctive features such as a grey hide with polygonal patterns, a typical 'saddle' on the nape, a slender head shape and a protrusion instead of a horn in females. The latter is a unique trait among Rhinocerotini species. In contrast, the Indian rhinoceros, a variable grazer, inhabits riverine grasslands in northern India and southern Nepal, displaying deep skin folds and tubercles. Ecological behaviours differ significantly, with the Sundaic rhinoceros being solitary wanderers and Indian rhinoceros forming temporary crashes. Both species possess unique adaptations for survival, emphasising the importance of understanding their systematics for effective conservation. The study further examines the interrelationships among the one-horned Asian species of the Rhinocerotidae family, highlighting their distinct features. The revision delves into skull morphology, dentition, and ecological dynamics, revealing evolutionary patterns and ancestral traits. Both single horned rhinoceroses went a separate and diverging way of evolution that was not triggered by geographical separation but by niche partitioning. Comparative analyses shed light on the evolutionary trajectory and ecological adaptations of each species. The fossils, the ecological and morphological adaptations of both species, suggest designating '*Rhinoceros*' *sondaicus* as distinct from *Rhinocerosunicornis*, under the one-horned rhinoceros *Eurhinoceros*, as proposed by Gray (1868). *Eurhinocerossondaicus* emerges as a persistently more primitive form.

## ﻿Introduction

“The forehead and the nose behind the base of the horn flat, both in the living animal and skull. *Eurhinoceros*.” With these words, John Edward Gray (1868: 1009) described a new one-horned rhinoceros to classify the Sundaic rhinoceros. A century later Heißig (1972) considered valid the genus *Eurhinoceros* to significantly distinguish *E.sondaicus* (Desmarest, 1822), a separate one-horned rhinoceros from *Rhinoceros* to which belongs the Indian rhinoceros *Rhinocerosunicornis* Linnaeus, 1758. Nardelli (1988: 41) foresaw that '*Rhinoceros*' *sondaicus* could be classified in a separate genus from *Rhinocerosunicornis*, suggesting that differences in their morphology and behaviour – such as the distinct external features of '*R.*' *sondaicus*’ head, especially its lips, and the fact that the former is a browser and the latter a grazer – support this distinction.

This work aims to examine the differing ecomorphological characters of *Rhinoceros* and *Eurhinoceros*. The results suggest that the single-horned rhinoceroses, *Rhinocerosunicornis* and *Eurhinocerossondaicus*, followed separate evolutionary paths not due to geographical isolation but rather as a result of niche partitioning. This can be followed by a number of stepping stones from the Middle Miocene onwards.

Heißig (1972: 29) reported the parallel presence of *Gaindatherium* and Eurhinocerosaff.sondaicus in the Middle Miocene Nagri formation of the Siwalik region of Pakistan. Fossil records of ancient rhinoceros species provide insights into their evolutionary history and the environmental conditions they inhabited (MacFadden 1998). By examining the morphology and geographic distribution of fossils, researchers can uncover patterns of niche partitioning and evolutionary divergence. Extant examples include the browser *Dicerosbicornis* Linnaeus, 1758 and the exclusive grazer *Ceratotheriumsimum* Burchell, 1817, which are sympatric today (MacFadden 1998: 282), as well as the variable grazer *Rhinocerosunicornis* and the generalist browser *Eurhinocerossondaicus* (Pandolfi et al. 2021: 9 and references therein as *Rhinoceros*), which had some overlapping ranges.

Pandolfi and Maiorino (2016) provide data on the coexistence of ‘Rhinoceros’ *sondaicus* and *Rhinocerosunicornis* in certain parts of their former ranges, with dietary specialisation playing a key role. The major distinctions between these two species are found in their dental morphology, indicating adaptation to different feeding strategies. *Eurhinocerossondaicus* is more adapted to browsing on softer, leafy vegetation, while *R.unicornis* is more inclined towards grazing on tougher, more abrasive plants material. This divergence in feeding habits minimised direct competition for resources, thus facilitating their coexistence.

Despite some similarities in cranial features, their teeth reflect evolutionary adaptations to different ecological niches, with *R.unicornis* showing adaptations for grazing-related diets and *E.sondaicus* for browsing. This separation allowed these species to share habitats without exhausting common food sources (Pandolfi and Maiorino 2016).

Extant species of Rhinocerotini Gray, 1821 (sensu Pandolfi 2015) exhibit diverse feeding habits that include grazing, mixed feeding, and browsing. Likewise, the diets of Pleistocene species were varied. In their study, Hernesniemi et al. (2011) analysed mesowear patterns in both extant and Pleistocene species, comparing *Dicerosbicornis*, *Ceratotheriumsimum*, *Dicerorhinussumatrensis* (Fischer, 1814), ‘*Rhinoceros’ sondaicus*, and *Rhinocerosunicornis* to fossil samples from Pleistocene Rhinocerotini, such as *Stephanorhinuskirchbergensis* (Jäger, 1839), *S.hemitoechus* (Falconer, 1859), *S.hundsheimensis* (Toula, 1902), and *Coelodontaantiquitatis* Blumenbach, 1799. When clustered by mesowear scores from the first and second molars, *Stephanorhinuskirchbergensis* grouped closely with *Dicerorhinussumatrensis*, while *S.hundsheimensis* showed affinity with ‘Rhinoceros’ sondaicus. These browsing species, together with the somewhat separate *Stephanorhinushemitoechus*, were distinctly separated from *Rhinocerosunicornis*, a variable grazer, which clustered near the grazer *Coelodontaantiquitatis* and the exclusive grazer *Ceratotheriumsimum*.

In the case of *Rhinocerosunicornis* and *Eurhinocerossondaicus*, the results of the present study imply that environmental pressures led to the development of differences in dental morphology, specialised feeding strategies, habitat adaptations and behavioural traits. Despite inhabited some overlapping areas, the two species evolved independently.

## ﻿The Sundaic rhinoceros *Eurhinocerossondaicus* (Desmarest, 1822)

### ﻿Distribution and subspecies

**Figure 1.**
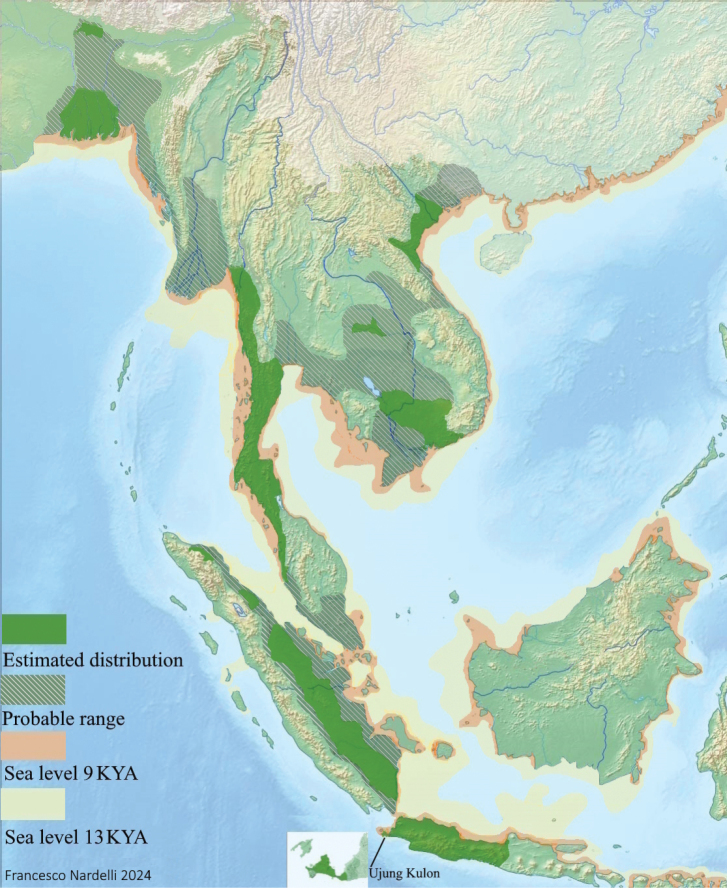
*Eurhinocerossondaicus*. Holocene (11500 BCE to present) distribution map based on reports and suitable habitats across space and time. Map based on Groves (1967: 224, fig. 1) and Rookmaaker (1980: 261, fig. 2); sea levels after Kim et al. (2023). Free vector and raster map data CC0 1.0.

The case of *E.sondaicus* distribution is a conundrum. If limited to the few human reports or artifacts, its range appears as fragmented patches throughout a vast area covering South and Southeast Asia. However, if inferred suitable habitats in the region of the documented locations are included, the range becomes much larger and contiguous (Fig. 1). The earliest known fossils of a *Rhinoceros* cf. ‘R.’ sondaicus in Southeast Asia are from central Myanmar, dating back to 9–8 million years ago (Longuet et al. 2024). The taxon has been traced from the Miocene (Heißig 1972) as Eurhinocerosaff.sondaicus and to the Pleistocene in northern Pakistan (Khan 2009; Abdul et al. 2014; Siddiq et al. 2016), Myanmar (Zin-Maung-Maung-Thein et al. 2006, 2010), Cambodia (Beden and Guérin 1973), Thailand (Suraprasit et al. 2016), Vietnam (Bacon et al. 2004), and Indonesia (Koenigswald 1935; Hooijer 1964; Antoine 2012). During the Holocene epoch, the species was probably widespread across various regions.

The presence of ‘Rhinoceros’ sondaicus has been confirmed in northeastern India and Bangladesh (Rookmaaker 1980, 1997, 2002), Myanmar (Loch 1937; Reynolds 1954), Thailand (McNeely and Cronin 1972), Cambodia (Poole and Duckworth 2005), Laos (Neese 1975), Vietnam (Loch 1937; Schaller et al. 1990), Malaysia (Sody 1959; Medway 1969), and Sumatra and Java (Sody 1959). Some reports on rhinoceroses from Bhutan exist, but actually pertain to *R.unicornis* (Rookmaaker 2016). The accounts originating from Hainan Island are unverified (Rookmaaker and Carpentier 2007). In Borneo, records of the species have been unclear (Rookmaaker 1977) or limited to a few fossils (Cranbrook 1986; Cranbrook and Piper 2007). The occurrence in southeastern China is uncertain (Rookmaaker 1980, 2006).

The Sundaic rhinoceros, also known as Javan or Lesser one-horned rhinoceros, is presently classified as 'Critically Endangered' by the International Union for Nature Conservation (IUCN). The species has been exterminated from most of its historical range, with only a small population of an unknown number, mostly males (Haryono et al. 2015; Nardelli 2016), confined within Ujung Kulon peninsula, the western tip of Java. In this location, it has become a ‘refugee’ species (Nardelli and Roboský 2023). Three subspecies have been recognised within the species *Eurhinocerossondaicus*. The nominal *E.s.sondaicus* (Desmarest, 1822) inhabited Peninsular Malaysia, Sumatra, Java (Sody 1959); *E.s.annamiticus* (Heude, 1892) was detected in regions of Thailand, Cambodia, Laos, Vietnam (the Indochina Peninsula; Groves and Guérin 1980), and eventually southeastern China (Rookmaaker 2006). *E.s.inermis* (Lesson, 1836) was identified in northeast India, Bangladesh (Rookmaaker 1997), and possibly Myanmar (Groves 1967). Some authors have suggested that these three taxa might be better treated as distinct phylogenetic species on biogeographical grounds (Gippoliti et al. 2013).

### ﻿Morphology

*Eurhinocerossondaicus* exhibits distinct characteristics that set it well apart from *Rhinocerosunicornis*. Only a few measurements of weight and size are available. Groves and Leslie (2011) indicate weight data with one female at 1500 kg, one male at 1200 kg and head-and-body lengths ranging from 305 to 344 cm, with shoulder heights between 120 and 170 cm. Today no statement can be made concerning any difference of body weight between the sexes. Their hide colour ranges from grey to dusky grey, making them easy to distinguish from *R.unicornis* that has brownish-grey coloration. Their body is covered by distinctive scale-like polygons that form a mosaic (Lydekker 1907; Peacock 1933; Harper 1945).

In contrast, the epidermal surface of *R.unicornis* exhibits limited patterns comprised of tubercles (Laurie et al. 1983) and its skin-folds differ from the arrangement typical of *E.sondaicus*: one behind the occiput close to the head, a transversal fold across the middle of the shoulders extending underneath the throat, another encompassing the entire body behind the shoulders and several folds that form distinctive patterns on the thighs and around the posterior. The posterior cervical fold extends over the nape of the neck, forming a distinctive, saddle-shaped shield, and serves as a diagnostic feature. Differently, the nape shield in *R.unicornis* is continuous. *Eurhinocerossondaicus* has a rugose integument, covered with a cuticle consisting of small, angular plates. Its head is characterised by distinctive nasal, frontal, and occipital bones and is narrower and longer compared to that of *R.unicornis*. These taxa also differ in the upper lip shape and length, reflecting their adaptation to different feeding habits, given that *E.sondaicus* is a generalist browser (Nardelli 2013) and *R.unicornis* is a variable grazer (Hullot et al. 2019). The upper lip is long and flexible, almost prehensile (Sody 1959). Griffiths (1993) also noted that the presence of the distinguishing 'saddle' on the neck was a manifestation of sexual dimorphism, although the data needed to confirm this hypothesis is lacking.

Even though the species is generally described as hairless, a sparse hairy covering has been documented by some authors (Groves 1967; Cave 1969; Nardelli and Robovský 2023). According to these records, body hair is more prominent in juveniles and gradually disappears with age, whereby adults only retain ear fringes, eyelashes, and tail bristles. The tail is fully exposed in side view, clearly protruding from its hind quarters (Groves and Leslie 2011). In females, the horn is a mere protrusion, allowing easy differentiation from males (Hoogerwerf 1970; Groves 1971). This sexual dimorphism in horn development is a unique characteristic of the only species among the Rhinocerotini where only males have a horn, averaging 20 cm in length. Even if the horn is nearly absent, the skull construction is adapted to bear a substantial horn also in females.

### ﻿Primary dental morphology

The strong lower incisors are directed almost straight forward (Fig. 4A). The cheek teeth are mesodont. The upper premolars are well molarised with a wide entrance in the central valley separating both lingual cusps (Fig. 6A [A]). On their labial side, there is no prominent metacone ridge, but the metacone is somewhat more lingually inclined like in molars (Fig. 6A [B]). The secondary folds are confined to the crochet, a crista is lacking (Fig. 6A [C]). On the lingual side, more or less complete cingula prevent lesions of the gingiva (Fig. 6A [D]). These observations were made on specimens of the Bavarian Zoological State Collection.

### ﻿Ecology

The behaviour of *Eurhinocerossondaicus* suggests a strong reliance on lowland forests (Nardelli and Robovský 2023) while mountainous excursions were likely situational or historically influenced by advancing anthropisation and competition with conspecifics at the time (Sody 1959 and references therein as *Rhinocerossondaicus*). *Rhinocerosunicornis* is documented to establish dominance hierarchies (Dinerstein 2003, 2011). In contrast, the study by Wilson (2021) indicates that both male and female ‘R.’ sondaicus primarily lead non-social lives. This solitary and retiring species, constrained to lowland areas, can still cover 15–20 km in a day. Nonetheless, most individuals occupy smaller patches for extended periods under favourable feeding conditions. Individual home ranges are not fixed for a lifetime and may shift based on the circumstances. A typical male’s territory can range from 12.5 to 21 square kilometres, whereas a female’s territory is smaller, spanning only 2.6–13.4 square kilometres (Amman 1986). Overall, female home ranges during seasonal periods are, on average, half the size of those of males (Setiawan et al. 2017). Female territories also overlap, allowing males to breed with multiple females. In contrast, male territories overlap only at the periphery (Hariyadi et al. 2016), resembling the behaviour of *D.sumatrensis*, also a browser and a rainforest dweller (van Strien 1985). Males are usually intolerant of each other, although sightings of two bulls wallowing together have been reported (Hoogerwerf 1970). They navigate a network of trails, not always continuous, linking wallows, pools, or river courses in which they swim (Sody 1959). Showing preference for easier routes, as a clear indication of adaptation to lowlands, they establish well-trodden tracks through thick scrub, while making detours around dense vegetation or steep slopes. In some cases, these tracks may turn into low tunnels shared with other animals.

Males as well as females often frequent wallows, approximately every two days, formed in depressions filled with rainwater that are concealed by vegetation, which can sometimes be quite deep and measure 20–35 m^2^. Although they show preference for fresh water, they are also attracted by muddy river banks and tidal forest margins. They often urinate while wallowing and this behaviour is assumed to be triggered by contact with water. This species is most highly active during night time and in the early morning, while resting during the day, especially around noon. As it needs to remain vigilant, it often rests in standing position, dozing with its head lowered and its ears constantly flicking (Nardelli 1988).

Aside from a few bamboo shoots, there is no evidence indicating feeding on grass. Its diet includes leaves from more than 200 plant species (Hariyadi et al. 2016). Occasionally, this foraging can be supplemented by browsing on rattan (*Calamus* spp.), palms (*Pandanus* spp.), young bamboos, mango, and fig fruit (Amman 1986).

Individuals mostly communicate through olfactory means, due to which they leave urine traces across their territory by squirting on vegetation. While males typically urinate in short, upward squirts—sometimes reaching up to 2 m, likely for dominance assertion and territory marking—females urinate in a continuous stream between their hind legs, scenting the ground. While moving along their trails, both males and females leave urine-scented mud from their wallows on the surrounding vegetation.

*Rhinocerosunicornis* and *Eurhinocerossondaicus* exhibit distinct behaviours for marking trails with secretions from their foot glands. The first scatters dung by kicking it around from their dung piles, whereas the latter, without forming piles, drags one hind foot through its faeces. Despite these differences, both species share the habit of scattering or dragging their hind feet through their dungs, leaving trails marked with glandular secretions that can extend for several meters (Hoogerwerf 1970; Amman 1986; Bhattacharya 2020b). The elusive nature of the species, combined with its remote and often inaccessible habitat, makes it challenging for researchers to observe and document the behaviours comprehensively. This indicates that a perception of *E.sondaicus* having a minimal range of vocalisations (Sody 1959 as *Rhinocerossondaicus*) could be attributed to their geographical isolation, small population size and the scarcity of research on the species (Wilson 2021 as *Rhinocerossondaicus*). Nevertheless, the analysis of video recordings from camera traps yielded insights into the vocalisations, identifying eight distinct call types. These recordings, comprising 196 individual calls, allowed the creation of detailed sonograms, a first for this species (Wilson 2021).

Although information on the breeding behaviour is scarce, based on the photographic and video evidence, it is identified that the females precede their calves akin to black rhinoceros *Dicerosbicornis*. Conversely, *R.unicornis* females follow theirs (Nardelli and Robovský 2023), sharing the behaviour of white rhinoceros *Ceratotheriumsimum* in Africa. Owing to this distinction, black rhinoceros calves are vulnerable to predation, particularly by spotted hyena *Crocutacrocuta* (Erxleben, 1777), whereas there are no recorded instances of white rhinoceros calves succumbing to the same predator (Hitchins 1986). Consequently, it is likely that in the past calves faced significant predation from tigers *Pantheratigris* (Linnaeus, 1758), which would have played a significant role in the species demise.

## ﻿The Indian rhinoceros *Rhinocerosunicornis* Linnaeus, 1758

### ﻿Distribution

The presence of *R.unicornis* in China and Southeast Asia during the Neogene and Quaternary epochs cannot be substantiated without more detailed fossil evidence. Reports from existing sources indicate that most of these fossils would be more accurately attributed to *Rhinocerossinensis* Owen, 1870 (Laurie et al. 1983; Yan et al. 2023) or ‘Rhinoceros’ sondaicus (Rookmaaker 1980, 2006).

**Figure 2.**
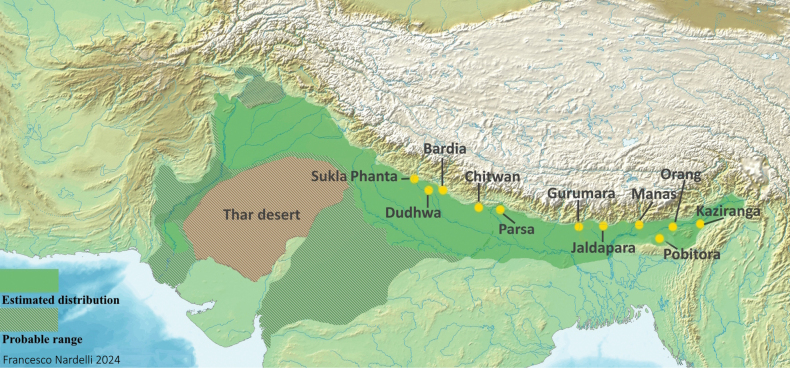
*Rhinocerosunicornis*. Holocene (11500 BCE to present) distribution map based on reports and suitable habitats across space and time. Yellow dots represent the current locations of *R.unicornis*. Map based on Rookmaaker (2000: 71, fig. 5). Free vector and raster map data CC0 1.0.

The *Rhinocerosunicornis* range covered the entire Indo-Gangetic plain: India, northern Bangladesh, Assam, Nepal, southern Bhutan, and north and eastern Pakistan with a few records from the south and one from Afghanistan (Choudhury 1985; Rookmaaker 2000). Today it is restricted to eleven main protected areas (Fig. 2) and listed 'Vulnerable' by the International Union for Nature Conservation with an in-situ population of ~ 4000 individuals. While Zschokke et al. (2011), Zschokke (2016), and Ghosh et al. (2022) highlighted genetic variability within the remaining populations of *R.unicornis*, there seems to be no recognised subspecies in the extant species.

### ﻿Morphology

*Rhinocerosunicornis* is considerably larger than *Eurhinocerossondaicus*, superseded in size only by the elephant and the white rhinoceros, with males weighing more than 2,000 kg and females reaching 1,600 kg. As one of the world’s largest land mammals, its shoulder height ranges from 160 to 190 cm and its length reaches nearly 400 cm (Laurie et al 1983). Adult males and females differ in only a few characteristics. Both sexes have a grey-brown hide and their skin folds have a pinkish hue. In contrast to *E.sondaicus* with five main skin folds, *R.unicornis* has four, which are more pronounced in males than in females. However, each individual has distinct skin fold patterns and raised tubercles on the hide; in fact these individual characteristics allowed Laurie (1978) to distinguish each of the animals in Royal Chitwan National Park in southern Nepal he studied between 1972 and 1976.

Males have significantly larger neck musculature and can also be distinguished from females by the distinctive deep folds behind and across the shoulders, on the neck, and before and across the thighs, while featuring a thick patch of skin on the upper part of the chest. Both males and females have a single horn ranging 20–40 cm in length, which is wider at the base in males.

### ﻿Primary dental morphology

The lower incisors are turned sideways (Fig. 4B). Their length is greater in adult males compared to females. The cheek teeth are subhypsodont, and block-like. The parastyle fold is reduced (Fig. 6B [A]), whereas a blunt, narrow metacone ridge is present on the labial side of the not-inclined metacone of the upper premolars (Fig. 6B [B]). In these teeth, the molarisation is not perfect, because the lingual cusps are still fused from the base to a varying height, leaving only a narrow slit between them in fresh premolars (Fig. 6B [C]). Crista and crochets are present and may unite to isolate a small medifossette from the central valley (Fig. 6B [D]). Lingual cingula are lacking or confined to small ridges (Fig. 6B [E]). These observations were made on specimens of the Bavarian Zoological State Collection.

### ﻿Ecology

The social structure differs in several aspects from that of *E.sondaicus*, including its semi-territorial behaviour, as individual superiority is asserted primarily through dominance (Laurie 1978, 1982; Dinerstein 2003), exhibiting complex intraspecific activities. These interactions can include dominance hierarchies, territorial disputes, mating behaviours, and social bonding. Factors such as age, sex, and individual temperament influence these interactions, leading to a diverse range of patterns within their populations (Bhattacharya 2020a). While usually solitary, individuals can occasionally form temporary groups at wallows or within seasonal feeding grounds. Some authors have observed crashes of up to a dozen rhinoceroses, predominantly sub-adults, wallowing simultaneously (Laurie 1978, 1982; Dinerstein 2003; Bhattacharya and Chakraborty 2016) and noted that they can sustain a higher population density in smaller areas than other Asian rhinoceroses (Hazarika and Saikia 2010). Research suggests that the species may possess some of the smallest annual and seasonal home ranges observed among mega-herbivores (Dinerstein 2011). Laurie (1978, 1982) also documented cases of young males being in close proximity to or lying near their elders while wallowing, but would be inevitably chased away after a while. Immature males tend to exhibit more gregarious behaviour than adults or immature females and young females might occasionally accompany an older cow and her calf. However, male calves spend a long time with their mothers or other sub-adult males due to their heightened vulnerability to attacks from adult males (Laurie 1982).

*Rhinocerosunicornis* has historically occupied various habitats, including marshes, alluvial plains, grasslands, and arid forests on the flood plains of major rivers such as the Indus, the Ganges, and the Brahmaputra, which they still share with elephants *Elephasmaximus* Linnaeus, 1758, and water buffaloes *Bubalusbubalis* (Linnaeus, 1758); (Laurie 1978, 1982; Laurie et al. 1983; Dinerstein 2003). They spend part of the day in water, especially during the hot and rainy monsoon season from June to September. Bathing not only helps them lower their body temperature and ward off biting insects, but also provides opportunities for socialising with other rhinoceroses. After exiting the water, they often rub their heads, necks, flanks, or horns against nearby trees, leaving mud deposits on the surrounding vegetation. The species exhibits a noteworthy behaviour by utilising communal defecation sites as they traverse their familiar habitats (Bhattacharya 2020b). Individuals of all age and sex groups in Chitwan National Park, Nepal, were observed using dung piles, as documented by Laurie (1978).

Laurie (1978, 1982) and Laurie et al. (1983) highlighted that rhinoceroses and hippopotamuses of the genus *Hexaprotodon* Falconer & Cautley, 1836, previously occupied similar ecological niches in India and although the latter have disappeared from India, the presence of *Hippopotamusamphibius* Linnaeus, 1758 in Africa, where they have asserted their dominance over water bodies, might explain why African rhinoceroses avoid swimming. They are most active during the night and tend to follow established trails linking salt licks, water sources and favoured foraging grounds. Individual ranges are not exclusive and are smaller than those of *E.sondaicus* and include areas covered by mixed pasture. Laurie (1978, 1982) documented ranges spanning from less than 0.5 km^2^ to nearly 9 km^2^. An adult would typically have a home range of ~ 3 km^2^, while sub-adults would have slightly larger ranges. Mature males in breeding condition show preference for establishing exclusive territories in the most fertile habitats, although no correlation between successful mating and territorial exclusivity has been established. In search of females, males may roam in several overlapping territories, each of approximately 6 km^2^, where the female population is the greatest. On the other hand, sub-adult males would seek areas less likely to be defended by dominant males. In contrast to mature *E.sondaicus* females, *R.unicornis* females cover more extensive ground and may contend for the most appealing settings.

*Rhinocerosunicornis* occupies a diverse habitat comprising grasslands, swamps, and riverine forests, where they graze on grasses and herbaceous plants from various families. They are highly flexible and adjust their diet not only to their habitat, but also to the grass season, especially during the monsoon rains, which increases their feeding resources.

The taxon is identified as a variable grazer, consuming 60–90% grasses with seasonal dietary shifts. Microwear patterns on its teeth show that, while primarily grass-eating indicated by the low complexity and high anisotropy on the grinding surfaces, it also processes a variety of plants material using its shearing surfaces (Hullot et al. 2019). Its lips are also well adapted to curl around short grasses, as well as allowing the stems of taller varieties to be pushed down, revealing the leaf blades. Laurie (1978, 1982) documented 183 plant species from 57 families in the dietary intake, 70–90% of which, depending on the season, is derived from 50 species of herbaceous plants. In addition to harvesting aquatic plants, a valuable source of sodium, by submerging their heads up to one meter underwater for 45 seconds at a time, they have been documented leaving hillside sal (*Shorearobusta*) forests to graze on grass shoots in newly burned areas. Tall grasses, those reaching up to 4–7 m in height, particularly *Pennisetumpurpureum*, as well as *Saccharum* species and grass of the genus *Narenga* are favoured sources of nutrition. They are abundant in the spring, but during the monsoon, shorter grasses are also consumed. The remaining portion of dietary intake comprises leaves or branches from shrubs, sedges, ferns, and aquatic plants.

Laurie (1982), and Dinerstein (2003, 2011) identified ten distinct vocalisations, each associated with specific behavioural contexts. Bhattacharya (2020b) further researched in depth vocal signals; though no sonograms analysis was performed.

### ﻿Genetic insights

This study briefly reports on the results of DNA analyses conducted separately on *E.sondaicus* and *R.unicornis* so far. While these studies provide valuable insights into the genetic makeup of each species individually, no direct comparative analysis has been performed between the two. As a result, specific genetic variations that could support the classification of these species into separate genera remain unconfirmed. The potential chromosomal differences between these taxa are reported here based on what is known from separate studies. While these discussions are informed by existing data, they are inherently speculative due to the lack of a direct comparative genetic analysis. Future research that includes direct molecular comparisons using consistent methodologies will be essential to define their evolutionary relationship more accurately.

Despite low genetic diversity being a long-term feature of rhinoceroses (Willerslev et al. 2009; Liu et al. 2021), the modern species exhibit the lowest levels of diversity, likely exacerbated by recent anthropogenic-driven population declines (Liu et al. 2021). The study by Margaryan et al. (2020) on '*Rhinoceros*' *sondaicus* identifies genetic diversity within the species, represented by historical sequences and extinct subspecies, emphasising the importance of preserving genetic diversity to maintain evolutionary potential. This study reveals fluctuations in genetic diversity over time, influenced by factors such as population size changes and environmental pressures. Examination of mitochondrial DNA (mtDNA) sequences from ancient specimens highlights evidence of recent lineage extinction events within populations (Margaryan et al. 2020). This suggests that certain genetic lineages or haplotypes present in ancestral communities are no longer found in recent ones, indicating a decline or loss in genetic diversity.

Due to the poor DNA quality of ancient specimens, e.g., ‘*Rhinoceros’ sondaicus*, Liu et al. (2021) could not assemble their genomes directly. Instead, they mapped them against other species genomes which, according to the authors, may introduce 'biases'. They further acknowledge the need for caution in interpreting results. Since only part of the Rhinocerotidae was studied, a considerable gap remains in understanding their evolutionary history until more comprehensive genome sequencing is conducted.

Genetic analysis of *Rhinocerosunicornis* reveals strong genetic structuring across different Indian states, with distinct genetic clades corresponding to specific geographical regions, suggesting limited gene flow and possible isolation mechanisms (Ghosh et al. 2022). Microsatellite analysis conducted by Ghosh et al. (2022) provides insights into genetic variation within populations. By analysing all frequencies and microsatellite loci, they identified specific genetic markers that exhibit variation across populations, indicating differences in genetic structure and connectivity. mtDNA analysis was also used by Ghosh et al. (2022) to infer the maternal evolutionary history and phylogeographic structure of extant populations.

**Figure 3.**
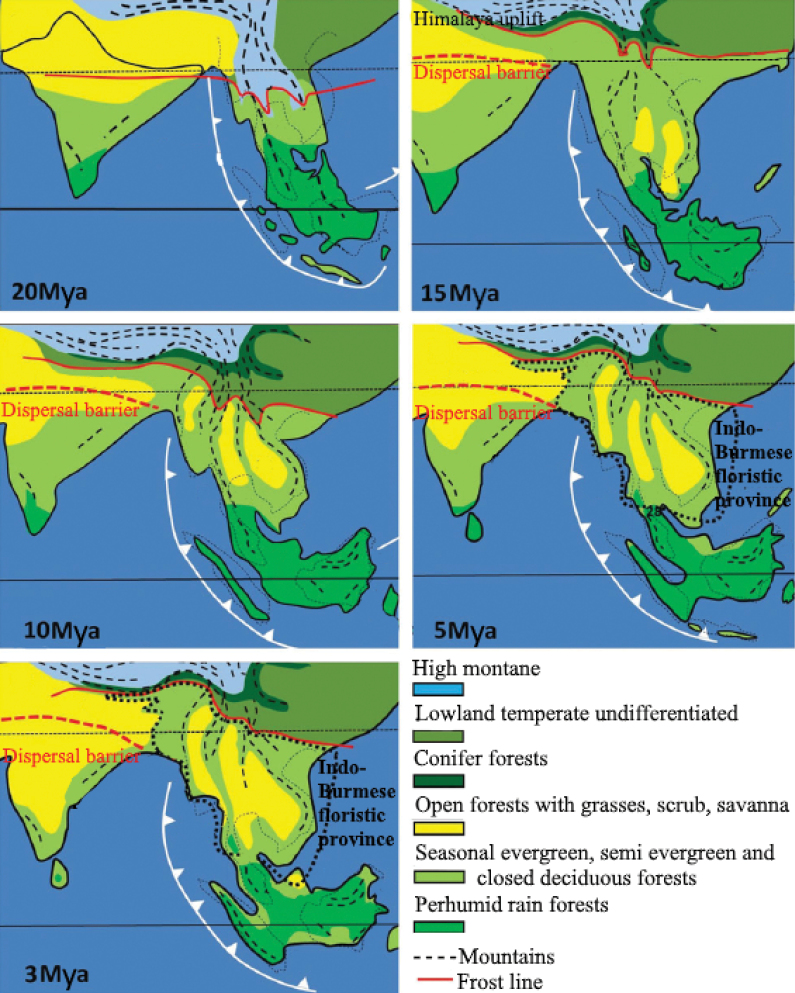
Palaeogeography, climate, and dispersals from the Early Miocene to the Late Pliocene. Adapted with permission from Morley (2018: 218).

Fossil evidence (Heißig 1972) suggests that the divergence of the *Rhinoceros* clade possibly took place during the Middle Miocene epoch 16–11.6 Mya. Some molecular studies estimate the divergence at 11.7–1.9 Mya (Tougard et al. 2001) and 13.4–13.2 Mya (Willerslev et al. 2009). Other studies, such as those by Margaryan et al. (2020) and Liu et al. (2021), calculate the divergence during the Pliocene 5.3–2.6 Mya (Fig. 3). During these periods, various factors such as changes in climate, habitat, and resource availability would have influenced the evolutionary trajectory of these taxa (Cerling et al. 1997; Longuet et al. 2023, 2024).

**Figure 4.**
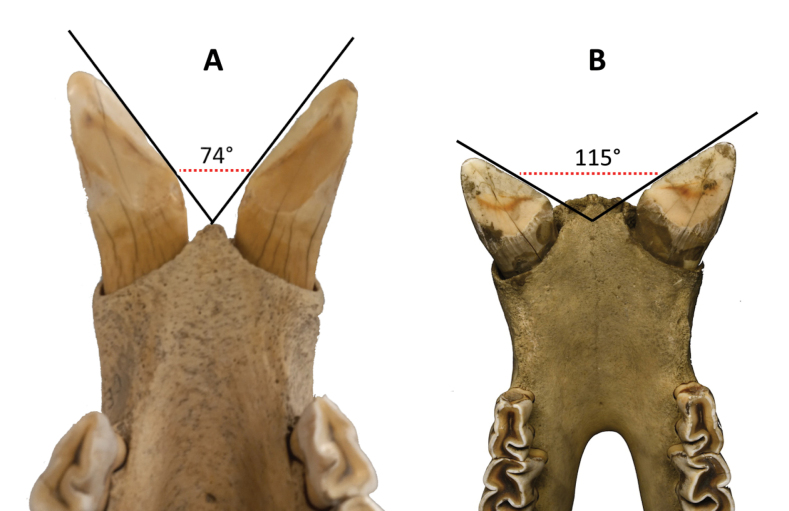
Lower incisors of **A***Eurhinocerossondaicus* and **B***Rhinocerosunicornis*. The images highlight a distinct difference in the angles and orientation of the incisors as they emerge from the mandible, reflecting specific characteristics. **A** Specimen no. 294 from the Museum of Natural History 'Giacomo Doria' of Genoa. Collected by G. B. Ferrari in 1873. Origin: Banten Province, Java. Photograph by Giuliano Doria **B** female specimen NHMUK ZD 1883•10•23•3 from the collections of the British Museum of Natural History, London. Collected by H.R.H. The Prince of Wales in 1883. Origin: Terai of Nepal. Photograph by Phaedra Kokkini.

**Figure 5.**
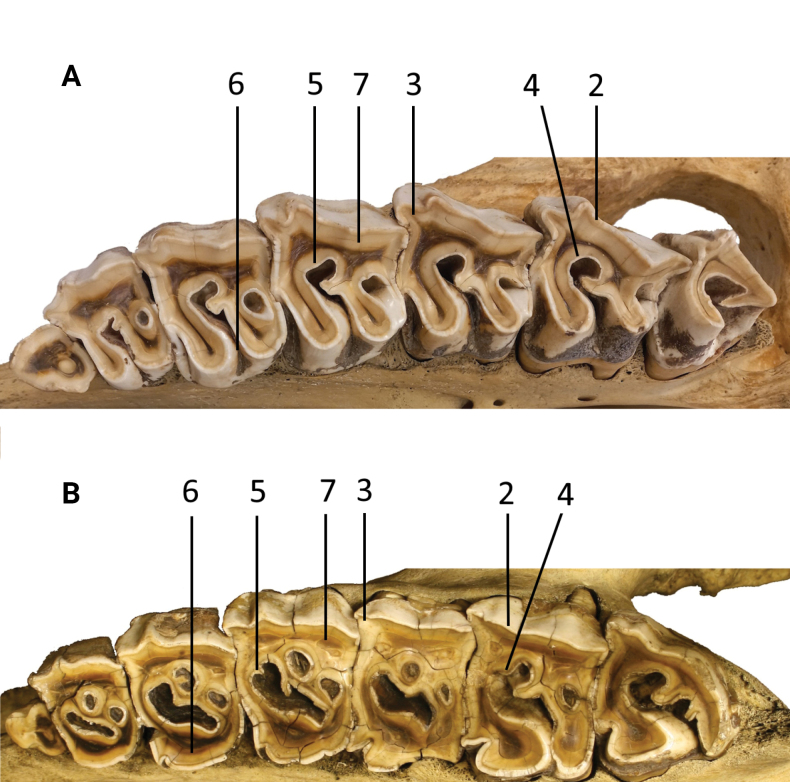
**A** Upper teeth of the left maxilla in occlusal view of *Eurhinocerossondaicus*, specimen no. 294, from the Museum of Natural History 'Giacomo Doria' of Genoa. Collected by G. B. Ferrari in 1873. Origin: Banten Province, Java. Photograph by Giuliano Doria **B** upper teeth of the left maxilla in occlusal view of *Rhinocerosunicornis*, specimen NHMUK ZD 1951•11•30•2, from the British Museum of Natural History, London. Collected by H. R. H. The Prince of Wales in 1883. Origin: Terai of Nepal. Photograph by Luca Pandolfi. Refer to Table 1 for numbered annotations.

**Figure 6.**
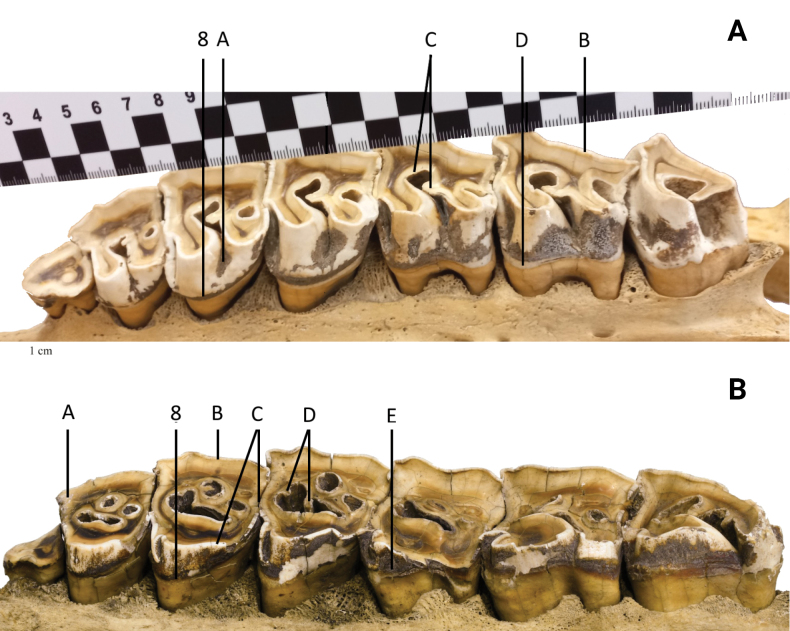
**A** Upper teeth of the left maxilla in lingual view of *Eurhinocerossondaicus*, specimen no. 294, from the Museum of Natural History 'Giacomo Doria' of Genoa. Collected by G. B. Ferrari in 1873. Origin: Banten Province, Java. Photograph by Giuliano Doria **B** upper teeth of the left maxilla in lingual view of *Rhinocerosunicornis*, specimen NHMUK ZD 1951•11•30•2, from the British Museum of Natural History Collections, London. Collected by H. R. H. The Prince of Wales in 1883. Origin: Terai of Nepal. Photograph by Phaedra Kokkini. Refer to Table 1 for numbered annotations.

## ﻿Findings and implications

“The key differences between *R.unicornis* and *R.sondaicus* are most apparent in their teeth, whereas the morphology of the skull is rather similar” (Pandolfi and Maiorino 2016: 10). Among the five extant species, the Asian ones stand out for possessing tusk-like incisors, a feature absent in African species. The synapomorphy of a sloped orbital floor connects these species without incisors. The three Asian forms can be categorised on a scale of increasing specialisation, ranging from *Dicerorhinussumatrensis* with two horns to *Eurhinocerossondaicus* and *Rhinocerosunicornis* with a single horn. The shape and posture of the skull align with this scale, but the same cannot be said for the morphology of the molars. While the molar and premolar type of *R.unicornis* can be understood as a high-crowned specialisation form of the primitive *D.sumatrensis*, the type of *E.sondaicus* diverges, showing similarities to African forms in the weakening of the metaconus rib, molarisation of the premolars, and strengthening of the inner cingulum (Heißig 1981).

The significance of the distinctions in cheek tooth morphology (Table 1) can be estimated only with a look at the paleontological evidence. Nearly all paleontological knowledge depends on the tooth morphology because skulls are rarely preserved. Cheek teeth with similarities to *Rhinoceros* and *Eurhinoceros* are known from Lower Miocene deposits in the Himalayan foreland basin (Forster-Cooper 1934). Other less complete specimens from the Lower Miocene Bugti beds, stored in the Natural History Museum, London show the fusion of lingual cusps more or less high above the enamel basis, the presence of a narrow metacone ridge in the upper premolars, and of both crista and crochet as in *Rhinoceros* (Forster-Cooper 1934: fig. 36). The first skulls with single horns and comparable tooth morphology are known from the Middle Miocene of Europe with *Lartetotherium* Ginsburg, 1974 and South Asia with *Gaindatheriumbrowni* Colbert, 1934, the latter with somewhat higher tooth crowns.

**Table 1.** Dental differences of upper cheek teeth in extant one-horned rhinoceroses (credit KH).

Character	*Rhinocerosunicornis*	*Eurhinocerossondaicus*
1. Crown height	subhypsodont	mesodont
2. Ectoloph of molars	flat	sinuous
3. Parastyle	short to reduced	long, prominent
4. Crista in molars	present	lacking
5. Crista in premolars	present	lacking
6. Lingual cusps of premolars	partly fused	widely separated
7. Metacone ridge of premolars	narrow, prominent, blunt	flattened and depressed
8. Lingual cingulum of premolars	vestigial or lacking	strong, sometimes continuous

The series with increasing tooth height continues to *Rhinocerossivalensis* Falconer & Cautley, 1847, which already has the typical skull profile of *Rhinoceros*. Differences in the combination of crown height, secondary folds, and cingulum formation show that this phylogenetic lineage was more complexly branched so that it is not easy to find the direct line to the living *Rhinoceros*. It makes no sense to distinguish primitive and progressive morphologies, as these are due to diverging dietary specialisations. Whereas *Eurhinoceros* was constantly a non-selective browser, the early relatives of *Rhinoceros* managed the transition from selective browsing to grazing, following the increasing dominance of grasses in the vegetation (Barry et al. 2002). As proposed by Singh et al. (2012), several sub-basins located south of the Himalayas experienced a gradual increase in aridity from approximately 12 million years ago to the present. This environmental shift likely has influenced the dietary habits of the rhinoceroses.

Owing to its confined habitat and the openness of its environment, as well as ex situ breeding programs, the ecological dynamics of *R.unicornis* are closely monitored and well documented. Extensive studies have been conducted on this species, yielding substantial data and allowing Laurie (1978, 1982), Laurie et al. (1983), Dinerstein (2003), and Bhattacharya (2020a) to provide comprehensive insights into the physical characteristics, socialisation, mating, feeding habits, and other patterns. Despite a fairly extensive body of literature on *E.sondaicus*, this species has not been given sufficient attention in investigations on the phylogenetic development of the Rhinocerotidae family. As a result, there is presently no consensus on their phylogenetic attributes. Several comparisons bring forth specific facts that would emerge from these studies. As shown in Tables 1, 2, *E.sondaicus* can be differentiated from *R.unicornis* based on several key characteristics of the teeth and the skulls as these features are more primitive in *E.sondaicus*, suggesting a harmonic developmental growth that uniformly impacts nearly all aspects of cranial and dental anatomy. According to Colbert (1942), this clear progression in the skull and tooth characters from *E.sondaicus* to *R.unicornis* is unique, allowing certain Pleistocene species of the one-horned rhinoceroses to assume intermediary positions between these two extant forms.

**Table 2.** Divergences in skulls between *R.unicornis* and *E.sondaicus* (after Colbert 1942: 2).

*Rhinocerosunicornis*	*Eurhinocerossondaicus*
1. Large and robust	1. Slenderer and lighter than *R.unicornis*
2. Nasals expanded into large, rounded horn boss	2. Less expansion in the nasals; horn boss pointed rather than rounded, very small in females
3. Occipital surface high and narrow. Skull deep	3. Occipital surface comparatively low and broad. Skull comparatively shallow
4. Deep 'saddle' in profile of skull between nasal and occipital vertex	4. Rather shallow 'saddle' in cranial profile
5. Zygomatic arch rounded at posterior termination	5. Zygomatic arch angular at posterior termination
6. Posterior margin of palate concave or with small median projection	6. Posterior margin of palate with median projection
7. Mesopterygoid fossa, basisphenoid and basi-occipital bones narrow	7. Mesopterygoid fossa, basisphenoid and basi-occipital bones comparatively broad
8. Pterygoids compressed and grooved	8. Pterygoids flattened and laterally expanded
9. Vomer thick and united to sides of pterygoid	9. Vomer thin, lamelliform, pointed and free
10. Premaxillaries broad	10. Premaxillaries relatively narrow

Based on skeletal morphology highlighted by current evidence in this study, *E.sondaicus* is considered the most primitive member of the one-horned rhinoceroses, exhibiting structural features that, through subsequent developments and adaptations became distinctive for Pleistocene species such as *Rhinocerossinensis*, *Rhinocerossivalensis*, and the contemporaneous *Rhinocerosunicornis* (Colbert 1942; Hooijer 1946: 675). *Eurhinocerossondaicus* is the sole Asian rhinoceros species well represented by a substantial number of specimens from Pakistan, Myanmar, Thailand, Cambodia, and Vietnam fossil deposits, allowing us to trace its evolutionary history from the Miocene (Heißig 1972) through the early Late Miocene (Longuet et al. 2024) to Plio-Pleistocene (Beden and Guérin 1973; Bacon et al. 2004; Zin-Maung-Maung-Thein et al. 2006, 2010; Khan 2009; Abdul et al. 2014; Suraprasit et al. 2016; Siddiq et al. 2016). In its dentition and skeletal characteristics, the representatives of Pleistocene *E.sondaicus* mirror the extant Sundaic rhinoceros.

Heißig’s (1981) cladistic analysis suggests that *R.unicornis* and *E.sondaicus* share a common ancestral lineage. However, the considerable diversity of fossil Rhinocerotini in South Asia, particularly in the Siwalik beds, presents challenges in attributing isolated remains to specific taxa, obscuring evolutionary inferences. Therefore, the relationship of fossil Rhinocerotini to extant species must be based primarily on dental characters. In most systematics studies, diet and cheek tooth morphology are often thought to be of less importance than other characters, even though dental microwear texture analysis can be employed to deduce ancient diets (Hullot et al. 2019: 398).

Following Heißig’s initial discovery (1972), more findings emerged to confirm the ancient presence of *Eurhinoceros* in the Siwalik beds of Pakistan. Khan (2009) examined several fossil rhinoceros species from the Pleistocene Siwalik beds, identifying the dental characteristics of some specimens as belonging to *Eurhinocerossondaicus* (as *R.sondaicus* and R.aff.sondaicus). Abdul et al. (2014) recorded the finding of Pleistocene rhinocerotid fossils from localities of Siwalik beds in the Gujrat district at Sar Dhok in Pabbi hills, where specimens belonging to *Rhinocerossivalensis*, ‘*Rhinoceros’ sondaicus*, and *Rhinocerosunicornis* were recovered as well as finds from Jari Kas in Mirpur district which belong to *Eurhinocerossondaicus* and *Rhinocerosplatyrhinus* Falconer & Cautley, 1847. These fossils, dating from the Tatrot and Pinjor stages of the Soan Formation ~ 3.5–0.9 million years ago include well-preserved maxillary and mandibular fragments, in addition to isolated teeth (Abdul et al. 2014). Siddiq et al. (2016) described fossil *Rhinoceros* aff. ‘*R.*’ *sondaicus* collected from various Siwalik bed localities, including the areas of Sar Dhok in Gujrat district, Tatrot in Jhelum district, Jari Kas in Mirpur district of Azad Jammu and Kashmir regions. These samples include maxillary and mandibular fragments, as well as isolated teeth.

Fossils of *Eurhinoceros* have been also found in Myanmar, Thailand, Cambodia, and Vietnam. Zin-Maung-Maung-Thein et al. (2006) identified right and left maxillae of ‘Rhinoceros’ sondaicus from the upper part of the Irrawaddy (Ayeyarwady) Formation. Additional cranial remains, including upper teeth, were recovered along the Irrawaddy sediments in central Myanmar (Zin-Maung-Maung-Thein et al. 2010). Fossil specimens of *Rhinoceros* cf. ‘R.’ sondaicus consisting of post-cranial remains were collected from the Tebingan area in the Magway Region of Myanmar (Longuet et al. 2024). The mammalian fauna from the Irrawaddy Formation is estimated based on the presence of several genera with well-established chronological distributions in the Siwalik deposits (Longuet et al. 2023, 2024). Deposits at Khok Sung in the Nakhon Ratchasima province of Thailand have preserved ‘*Rhinoceros’ sondaicus* cranial, mandibular, and dental fossils (Suraprasit et al. 2016). At the Phnom Loang, Middle Pleistocene to the Holocene site in Cambodia, Guérin (1973) identified ‘*Rhinoceros’ sondaicus guthi* Guérin, 1973 fossil specimens. Molar fossils provisionally referred to ‘Rhinoceros’cf.sondaicus, were identified at the Pleistocene Ma U'Oi cave, northern Vietnam (Bacon et al. 2004). In the above-mentioned studies four taxa were identified based on dental morphology and dimensions. Fossils of Rhinocerotini occur as early as in the Lower Miocene of the Bugti beds. Some of these single finds, stored in the Museum of Natural History, London are still undescribed.

The differences, especially in the premolars, can be associated with the dissimilar diets of *R.unicornis* and *E.sondaicus* (Hullot et al. 2019). *Rhinocerosunicornis* is a variable grass eater and has high-crowned, block-like upper cheek teeth that can withstand, for a lifetime, the abrasion caused by fibres and silicic acid. On the other hand, *E.sondaicus* is a generalist browser, exposing its premolars to twig fragments, requiring cingula to prevent lesions of the gums. Without the stress of strong abrasion, the teeth could stay mesodont. The premolars possess a narrow metacone rib and a high lingual wall, even if the lingual cusps are separated by lingual and labial furrows. A lingual cingulum may be present or absent, and this stage of molarisation was defined as 'paramolariform' by Heißig (1969: 16). It is rather widespread in the tribe Rhinocerotini and still present in the living *R.unicornis*.

Members of the *Rhinoceros* clade are well represented in the Siwalik beds, starting with the genus *Gaindatherium* which is characterised by the absence of a lingual cingulum and a distinct metacone rib. This lineage appears to transition into the higher-crowned *Rhinocerossivalensis* and eventually leads to the even more high-crowned extant species, although earlier and contemporaneous specimens of *R.sivalensis* appear to be too high-crowned to be considered direct ancestors of *Eurhinoceros*. *Eurhinoceros* seems to have arrived relatively early in Java during the Pleistocene, potentially with an ancestor referred to as ‘*Rhinoceros’ sivasondaicus* Dubois, 1908. Evidence of this evolutionary pathway can be traced through fossil specimens found at Ngandong, Sangiran, Djetis, and Trinil Pleistocene localities in Java (Koenigswald 1935; Hooijer 1964; Antoine 2012).

Looking for earlier members of the *Eurhinoceros* lineage in the Siwalik collections, Heißig (1972: 29) described two premolars from Sethi Nagri and an upper molar and some lowers from two localities of the Chinji Formation in Pakistan. The specimens come from Dehm’s expeditions to the Siwalik Hills in Pakistan (winter 1955/1956), housed in the Bavarian State Collection of Paleontology and Geology, Munich and the University of Utrecht Collection, Netherlands. They all differ from *Gaindatherium* by the formation of cingula. The complete set of characters foreshadowing the morphology of the recent *Eurhinoceros* shows the upper premolar described by Heißig (1972: 18) from the Dhok Pathan Formation of Parlewali. It differs from the premolar figured by Matthew (1929: figs 33–34) with the probable provenance Dhok Pathan by the loss of the metacone rib, the deeper separation of the lingual hills and the formation of a lingual cingulum around the protocone base.

The two premolars were identified as Eurhinocerosaff.sondaicus due to their resemblance to the extant species. Heißig (1972: 30) detailed a M2 and two m3, one of them on a mandible fragment from the Middle Miocene of the Chinji Formation as a probable *Eurhinoceros* sp. as illustrated by Heißig (1972: pl. 5, figs 8, 9; pl. 7, figs 1, 2). The upper molar, albeit somewhat fragmentary, aligns with the features of the extant species in the faint antecrochet and the subtle protocone constriction, typical traits of most Rhinocerotini. Additionally, it shares the development of a lingual cingulum and the absence of a metacone rib. The absence of the cingulum in the external groove of the lower molars is also not observed in all living Rhinocerotini e.g., *Diceros* and *Dicerorhinus* (Heißig 1972: 30). If this determination is correct, these specimens would prove a separation of the one-horned rhinoceroses from the main stock of Rhinocerotini around the Middle Miocene.

At first sight, *R.unicornis* and *E.sondaicus* display similarity in traits such as the head position and the partly or nearly complete armour of the skin, although both are distinctly different when analysed in depth. The relatively high position of the head in the variable grazer *R.unicornis*, compared to the exclusive grazer *C.simum*, as shown by the forward inclination of the occipital plane, corresponds to its feeding habits, which include consuming very tall elephant grass (*Pennisetumpurpureum*) and reaching for high-hanging twigs in dense forest vegetation (Bales 1996: 274). The armour, however, is not easily understood as convergence and may have been a characteristic of shared ancestral lineage. According to Endo et al. (2009), the development of skin folds in *R.unicornis* is considered a thermoregulatory adaptation, serving to protect the rhinoceroses from heat.

The morphology of the skull shows a strong correlation with the hypsodont index (Piras et al. 2010). This indicates that adaptations to different feeding habits occur deeper within the rhinoceros evolutionary tree, rather than limited to the species level. These adaptations include distinct modifications to the posterior part of the skull, which is known to vary with feeding behaviour: while the mandible and skull are more constrained by phylogeny and their developmental integration, the upper tooth row is less influenced by this relationship and teeth are the most adaptable cranial structure in evolutionary terms (Piras et al. 2010).

The skull dimensions in adults of the two living species are as follows: the occipito-nasal distance range is 613–694 mm in *unicornis* and 567–669 mm in *sondaicus*; the maximum width at the zygomatic arches is 355–435 mm in *unicornis* and 324–365 mm in *sondaicus*; *unicornis* has a mandibular length of 526–600 mm and a condylar height of 277–309 mm, while *sondaicus* has a mandibular length of 467–518 mm and a condylar height of 208–247 mm (Guérin 1980). At this point, it is practical to delineate the disparities in the skull between the two extant species (Table 2). These distinctions carry significant importance for prospective discussions regarding the phylogenetic position of each taxon in relation to the other (Colbert 1942).

**Figure 7.**
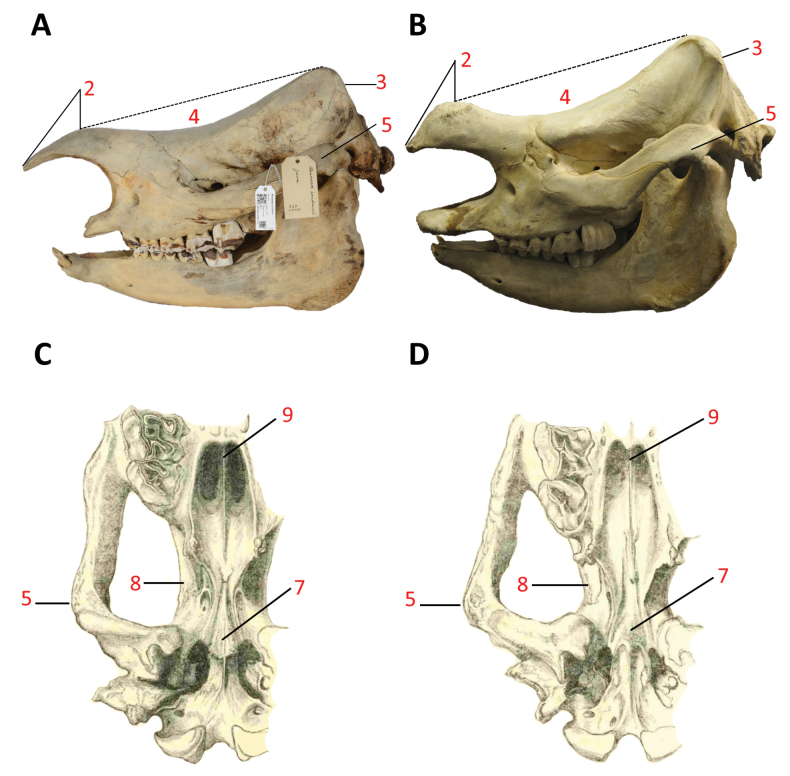
**A** Left side view of the skull of *Eurhinocerossondaicus* (juv.), specimen NHM-DMA-26801/1, collected in Java, 1838. Credit: Natural History Museum, Oslo. Adapted from Matschiner (2021: 4842), with permission from Elsevier Eds. Photograph by Lars Erik Johannessen **B** left side view of the skull of *Rhinocerosunicornis* (juv.), Smithsonian NMAH. Licensed under CC BY-SA 4.0. The image has been modified by isolating the skull and inverting its position horizontally. Photograph by David J. Stang **C** partial ventral view of the cranium of *Eurhinocerossondaicus***D** partial ventral view of the cranium of *Rhinocerosunicornis*. Adapted from Flower (1876: 446, 447). Main cranial differences outlined following Colbert (1942). Refer to Table 2 for numbered annotations.

The skull morphology of the two genera differs, with *R.unicornis* having, in proportion, a larger and heavier skull compared to *E.sondaicus*. Additionally, the occiput of *R.unicornis* is higher and narrower, making the dorsal outline of the skull very concave. Overall, while both genera share certain anatomical features, they also exhibit distinct differences in skull morphology (Fig. 7). The analysis of one ‘*Rhinoceros’ sondaicus* specimen from the Manchester Museum, UK by Cave (1985) reveals several key features that are characteristic of *E.sondaicus* cranial morphology. The occipital plane is inclined forward. The orbital-aural length is longer than the orbito-nasal dimension. This ratio of orbital-aural length to orbito-nasal dimension is characteristic of *E.sondaicus* and contributes to its unique cranial proportions. The infra-orbital foramen is positioned above the second premolar tooth, which is a characteristic feature of its cranial anatomy. The mode of anterior attachment of the petro-sphenoidal ligament serves as a distinguishing feature between *E.sondaicus* and *R.unicornis*. In *E.sondaicus*, the anterior attachment of the petro-sphenoidal ligament is characterised by a small tubercle located at the termination of the Eustachian crest. This tubercle serves as the point of connection for the petro-sphenoidal ligament. On the other hand, in *R.unicornis*, there is no corresponding tubercle at the termination of the Eustachian crest. Instead, the petro-sphenoidal ligament attaches subtly to the posterior free margin of the alisphenoid bone. This difference in the anterior attachment of the petro-sphenoidal ligament between the two taxa is a significant anatomical distinction that aids in the identification and classification of their respective crania (Cave 1985). By examining these features, it is possible to differentiate between their specimens and gain insights into the unique cranial morphology of each species.

**Figure 8.**
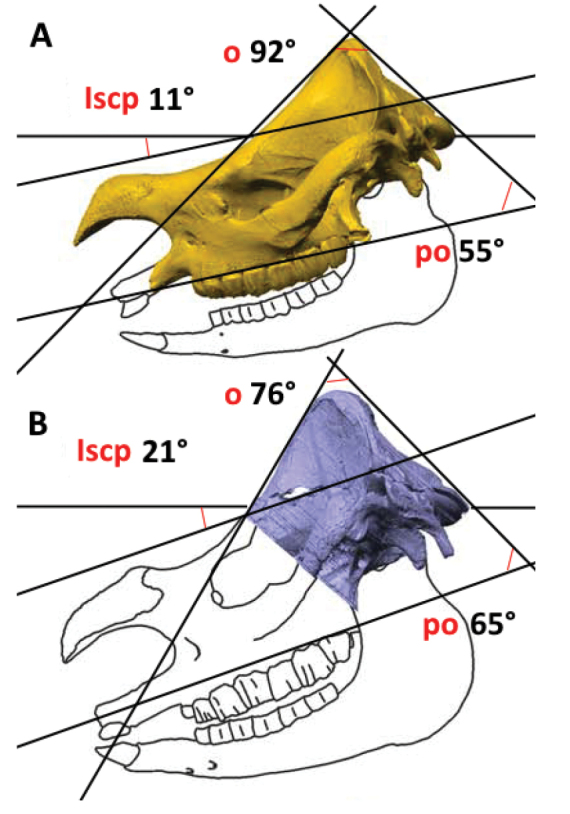
Polygonal surface model of **A***Eurhinocerossondaicus* skull (in yellow) and **B***Rhinocerosunicornis* skull (in blue) with measured angles. Abbreviations: **Iscp**–angle between plane spanned between the lateral semicircular canals (LSC) of both inner ears and the palatal plane; **o**–angle of the occipital crest between the occipital plane and the parietal plane; **po**–angle between occipital plane and palatal plane. Skull drawings and colours have been maintained from the original. Adapted with permission from Schellhorn (2018: 53).

“The (skulls) shape in the lateral view reflects ecological niche, in particular feeding type from browsing to grazing, and it also represent taxonomic discrimination” (Pandolfi et al. 2021: 1). The lateral shape of rhinoceros skulls plays a crucial role in reflecting their ecological niches, particularly distinguishing feeding habits ranging from browsing to grazing. Browsing species, typically exhibit different skull structures compared to grazing species. In the lateral view, the shape helps identify these feeding behaviours by highlighting morphological traits adapted to their diets, such as the orientation of the occipital area and the development of other cranial features. This differentiation in skull forms also serves as an important indicator of taxonomic distinctions among rhinoceros species, aiding in the classification and understanding of their evolutionary adaptations. These anatomical differences are consistent with the feeding habits, as grazing rhinoceroses like the white and Indian rhinoceroses feed closer to the ground, while browsing species such as the Sundaic and the Asiatic two-horned rhinoceroses feed on leaves and saplings. This connection between skull morphology and dietary preferences can also be traced through fossil records, demonstrating how rhinoceroses adapted to varying environments and food sources over time (Pandolfi et al. 2021). As shown in Fig. 8, a smaller angle **o** suggests a posteriorly extended occipital crest, which corresponds to a downward-oriented skull posture. When the lateral semicircular canals (LSC) are aligned horizontally, the palatal plane in *E.sondaicus* is nearly horizontal (Fig. 8A), whereas in *R.unicornis*, the rostrum and palatal plane tilt slightly downward towards the ground (Fig. 8B). The angle between the plane defined by the LSC of both inner ears and the palatal plane is also smaller in *E.sondaicus*.

Schellhorn (2018) highlights that the connection between the shape of the occiput and head posture has been recognised for a long time (Zeuner 1934; Bales 1996). Zeuner (1934) was the first to quantitatively analyse the shape of the occiput, specifically the angles **o** and **po**, and found that the angle **o** that is formed between the parietal and occipital planes is smaller in grazing rhinoceroses compared to browsing species. Schellhorn (2018: 58) also affirms: “These results also support the hypothesis that rhinoceroses with large lower second incisors but one small nasal horn, like *R.sondaicus*, have a horizontal head posture”.

Heißig (1972, 1989) suggested that fossil rhinoceroses with strong lower tusks likely used them as defensive weapons against predators, similar to the behaviour of extant Asian rhinoceroses. This inference is based on the well-documented behaviour of the living species, which use their tusks in combat (Sody 1959; Owen-Smith 1988). In contrast, the African species, which have completely reduced incisors, rely on their large horns for fighting (Dinerstein 2011). Dinerstein (2011) further proposed a potential link between these behaviours and typical head posture, noting that rhinoceroses with strong lower tusks might favour a horizontal head posture, which would facilitate the use of their tusks as weapons.

This idea is reinforced by the observation that a downward head posture is predominantly seen in both living and fossil rhinoceroses with large horns and without lower second incisors (Heißig 1973). Schellhorn (2018) and Benoit et al. (2020) emphasise the intricate relationship between anatomical features, such as the LSC orientation and occipital shape, and behavioural aspects like feeding preferences and head posture. While Benoit et al. (2020) underline the complexity and potential limitations in reconstructing these features accurately in extinct species, Schellhorn (2018) demonstrates a practical application in extant rhinoceroses, providing new insights that could potentially be adapted to fossil records. A pivotal insight from the work of Benoit et al. (2020) is the pronounced influence of phylogenetics on all examined variables in ungulates. Their findings propose that, in ungulates, LSC orientation predominantly corresponds to evolutionary relationships. For a more precise understanding, upcoming research endeavours should scrutinise each subclade independently, demanding a broader sample size for each (Pandolfi et al. 2021).

## ﻿Conclusions

The one-horned Asiatic rhinoceroses are examples of evolutionary histories driven by ecological pressures. Adaptations of large terrestrial mammals to various environments are linked to the diversity of food items they can consume, which is reflected in the variation of their dental and cranial morphologies. In rhinoceroses, these adaptations are identified in their teeth structure and head posture. Evidence on feeding habits aligns with the positioning of skulls within the morphospace, allowing us to infer the existence of distinct feeding types or ecomorphotypes.

Ecological niche modelling studies have demonstrated differences in habitat preferences and ecological requirements, suggesting niche partitioning as a mechanism for coexistence and evolutionary divergence. These models predict distinct distributions and habitat suitability for each taxon also within overlapping geographical ranges.

Behavioural observations of *R.unicornis* and *E.sondaicus* in their natural habitats have provided insights into their substantial dissimilar dietary intake, marked activity patterns and distinct habitat utilisation. These observations indicate specific behaviours supporting the notion of niche partitioning to reduce competition. This separation of ecological niches not only prevented direct competition but also contributed to their distinct evolutionary trajectories. The fossils support this divergence, with evidence indicating that *E.sondaicus* evolved to exploit a browsing niche, while *R.unicornis* became increasingly specialised as a grazer. The significant distinctions in tooth morphology, including the variation in wear patterns, reflect their adaptation to different diets and ecological settings over extended geological timescales.

Genetic assessments were not feasible in this study due to the lack of comparative genetic research. Studies on population dynamics and demographic history reveal patterns of genetic diversity, gene flow, and population structure within *R.unicornis* and *E.sondaicus* populations.

The morphological and ecological differences between the two taxa are not merely superficial adaptations to different dietary intakes, but significant structural changes that have evolved over paleontological epochs. The distinctions reflect deep evolutionary adaptations, not short-term ecological plasticity. The study identifies key divergences, not minor traits but fundamental anatomical features tied to their evolutionary adaptations. These diversities are expected to be satisfactory in taxonomy to justify genus-level distinctions, as seen in other genera like *Ceratotherium* and *Diceros*. Since the extant African rhinoceroses are classified into different genera, it is reasonable to separate *Rhinoceros* and *Eurhinoceros* due to their similar dietary adaptations, among a number of other characteristics. By integrating evidence from paleontological records, ecological niche modelling, morphology, behavioural observations and population dynamics, this assessment substantially supports the idea of distinct evolutionary trajectories for *Rhinocerosunicornis* and *Eurhinocerossondaicus*.

Available data do not provide justification for classifying *Eurhinocerossondaicus* as a congeneric species with *Rhinocerosunicornis* or as a subgenus within *Rhinoceros*. In our view, the phenotypic and adaptive differences observed between the two lineages warrant a reassessment of its taxonomic status at the genus level. This approach not only reflects their evolutionary separation but also provides a clearer framework for better understanding of their distinct characteristics within a phylogenetic context.

## ﻿Additional information

### Author ORCIDs

Francesco Nardelli https://orcid.org/0000-0001-9317-5376

### Data availability

All of the data that support the findings of this study are available in the main text.

## References

[B1] Abdul AM Cerdeño E Akhtar M Khan MA Siddiq Kh (2014) An account of the Upper Siwalik rhinocerotids of Pakistan. Abstract volume of the 4th International Palaeontological Congress, Mendoza, Argentina, 549 pp.

[B2] Amman H (1986) Contributions to the ecology and sociology of the Javan rhinoceros (*Rhinocerossondaicus*, Desmarest). Basel University, Inaugural Dissertation.

[B3] Antoine P-O (2012) Pleistocene and Holocene rhinocerotids (Mammalia, Perissodactyla) from the Indochinese Peninsula. Comptes Rendus Palevol, Elsevier 11: 159–168. 10.1016/j.crpv.2011.03.002

[B4] Bacon AM Demeter F Schuster M Vu TL Nguyen KR Antoine P-O Sevket S Ha HN Nguyen MH (2004) The Pleistocene Ma U’Oi cave, northern Vietnam: palaeontology, sedimentology and palaeoenvironments. Geobios 37: 305–314. 10.1016/j.geobios.2003.03.010

[B5] Bales GS (1996) Skull evolution in the Rhinocerotidae (Mammalia, Perissodactyla): Cartesian transformations and functional interpretations. Journal of Mammalian Evolution 3(3): 261–279. 10.1007/BF01458183

[B6] Barry JC Morgan ME Flynn LJ Pilbeam D Behrensmeyer AK Raza SM Khan AI Badgley C Hicks J Kelley J. (2002) Faunal and environmental change in the late Miocene Siwaliks of northern Pakistan. Paleobiology 28 (S2): 1–71. 10.1666/0094-8373(2002)28[1:FAECIT]2.0.CO;2

[B7] Beden M Guerin C (1973) Le gisement des vertébrés du Phnom Loang (Province de Kampot, Cambodge). Travaux et Documents de l’ORSTOM 27: 3–97.

[B8] Benoit J Legendre LJ Farke AA Neenan JM Mennecart B Costeur L Merigraud S Manger PR (2020) A test of the lateral semicircular canal correlation to head posture, diet and other biological traits in “ungulate” mammals. Scientific Reports 10: 1–21. 10.1038/s41598-020-76757-0 33177568 PMC7658238

[B9] Bhattacharya A (2020a) Ecology, behaviour and management practices of Great Indian one Horned Rhinoceros at Gorumara, Jaldapara and Kaziranga national parks, India. Thesis presented to Raiganj University.

[B10] Bhattacharya A (2020b) Communication types in Indian Rhinoceros (*R.unicornis*, Linnaeus). International Journal of Trend in Research and Development 7(4): 188–194.

[B11] Bhattacharya A Chakraborty K (2016) Defecation behaviour of great Indian one horned rhinoceros (*R.unicornis*, Linnaeus). International Journal of Science and Research 5(7): 923–978.

[B12] Blumenbach JF (1799) Handbuch der Naturgeschichte. Sechste Ausgabe (6^th^ ed). Johann Christian Dieterich, Goettingen, 703 pp.

[B13] Burchell WJ (1817) Ueber eine neue Gattung Nashorn. Isis (Oken) 1: 1318–1319. [figs 1, 2]

[B14] Cave AJE (1969) Hairs and vibrissae in the Rhinocerotidae. Journal of Zoology, London 157(2): 247–257. 10.1111/j.1469-7998.1969.tb01700.x

[B15] Cave AJE (1985) An unrecorded specimen of the Javan rhinoceros (*Rhinocerossondaicus*). Journal of Zoology, London 207: 527–535. 10.1111/j.1469-7998.1985.tb04949.x

[B16] Cerling T Harris J MacFadden B Leakey MG Quade J Eisenmann V Ehleringer J.R (1977) Global vegetation change through the Miocene/Pliocene boundary. Nature 389: 153–158. 10.1038/38229

[B17] Choudhury A (1985) Distribution of Indian one-horned rhinoceros (*Rhinocerosunicornis*). Tiger Paper 12(2): 25–30.

[B18] Colbert EH (1934) A new rhinoceros from the Siwalik beds of India. American Museum Novitates 749: 1–13. 10.70249/9798893981964-002

[B19] Colbert EH (1942) Notes on the Lesser one-horned rhinoceros, *Rhinocerossondaicus*, 2. The position of *Rhinocerossondaicus* in the phylogeny of the genus *Rhinoceros*. American Museum Novitates 1207: 1–5.

[B20] Cranbrook Earl of (1986) A review of fossil and prehistoric remains of rhinoceroses of Borneo. Sabah Museum and Archives Journal 1(1): 50–110.

[B21] Cranbrook Earl Piper PJ (2007) The Javan rhinoceros *Rhinocerossondaicus* in Borneo. Raffles Bulletin of Zoology 55(1): 217–220.

[B22] Desmarest AG (1822) Mammalogie, ou description des espèces des Mammifères. Veuve Agasse, Paris, 277–555.

[B23] Dinerstein E (2003) The return of the unicorns: natural history and conservation of the Greater one-horned rhinoceros. Columbia University Press, New York, 316 pp. 10.7312/dine08450

[B24] Dinerstein E (2011) Family Rhinocerotidae (Rhinoceroses). In: Handbook of the mammals of the world, vol. 2: Hoofed mammals. Lynx, New York, 144–181.

[B25] Dubois E (1908) Das geologische Alter der Kendeng-oder Trinilfauna. Tijdschrift van het Koninklijk Nederlandsch Aardrijkskundig Genootschap (2)25: 1235–1270.

[B26] Endo H Kobayashi H Koyabu D Hayashida A Jogahara T Taru H Oishi M Itou T Koie H Sakai T (2009) The morphological basis of the armor-like folded skin of the Greater Indian Rhinoceros as a thermoregulator. Mammal Study 34(4): 195–200. 10.3106/041.034.0403

[B27] Erxleben JCP (1777) Systema regni animalis per classes, ordines, genera, species, varietates, cum synonymia et historia animalium. Weygandianis, 636 pp. 10.5962/bhl.title.15933

[B28] Falconer H Cautley PT (1847) Fauna antiqua sivalensis, being the fossil zoology of the Sewalik Hills, in the North of India Illustrations, part VIII: Suidae and Rhinocerotidae. Smith, Elder and Company, London, pls 69–80. 10.5962/bhl.title.61447

[B29] Falconer H (1859) [In Gaudin CT (Ed.)] Modifications apportés par Mr. Falconer a la faune du Val d’Arno. Bulletin de la Société Vaudoise des Sciences Naturelles 6(44): 130–131.

[B30] Falconer H Cautley PT (1836) Note on the fossil hippopotamus of the Siwalik Hills. Asiatic Researches 19(3): 39–53.

[B31] Flower WH (1876) On some cranial and dental characters of the existing species of rhinoceroses. Proceedings of the Zoological Society of London 1876 May 16: 443–457. 10.1111/j.1096-3642.1876.tb02583.x

[B32] Forster-Cooper C (1934) The extinct Rhinoceroses of Baluchistan. Philosophical Transactions of the Royal Society of London (B) 223: 569–616. 10.1098/rstb.1934.0013

[B33] Ghosh T Kumar S Sharma K Kakati P Sharma A Mondol S (2022) Consideration of genetic variation and evolutionary history in future conservation of Indian one-horned rhinoceros (*Rhinocerosunicornis*). BMC Ecology and Evolution 22(92): 1–13. 10.1186/s12862-022-02045-2 35858827 PMC9301832

[B34] Ginsburg L (1974) Les Rhinocérotidés du Miocène de Sansan (Gers). Compte Rendu des Séances de l’Académie des Sciences, Paris 278 (5) D: 597–600.

[B35] Gippoliti S Cotterill FPD Groves CP (2013) Mammal taxonomy without taxonomists: a reply to Zachos and Lovari. Hystrix Italian Journal Mammalogy 24: 145–147.

[B36] Gray JE (1868) Observations on the preserved specimens and skeletons of the Rhinocerotidae in the collection of the British Museum and Royal College of Surgeons, including the description of three new species. Proceedings of the Zoological Society of London: 1003–1032.

[B37] Griffiths M (1993) The Javan rhino of Ujung Kulon: an investigation of its population and ecology through camera trapping. Directorate General of Forest Protection and Nature Conservation and the World Wide Fund for Nature Indonesia Program, Jakarta, 1–92.

[B38] Groves CP (1967) On the rhinoceroses of South-East Asia. Säugetierkundliche Mitteilungen 15(3): 221–237.

[B39] Groves CP (1971) Species characters in rhinoceros horns. Zeitschrift fur Saugetierkunde 36(4): 238–252. [figs 1–22]

[B40] Groves CP Guérin C (1980) Le *Rhinocerossondaicusannamiticus* d‘Indochine: distinction taxinomique et anatomique; relations phylogénétiques. Geobios 13(2): 199–208. 10.1016/S0016-6995(80)80028-3

[B41] Groves CP Leslie Jr DM (2011) *Rhinocerossondaicus* (Perissodactyla: Rhinocerotidae). Mammalian Species 43(887): 190–208. 10.1644/887.1

[B42] Guérin C (1973) Rhinocerotidae: 19–50, pls. 1–4. In Beden M and Guérin C (1973) Le gisement des vertébrés du Phnom Loang (Province de Kampot, Cambodge). Travaux et Documents de l‘ORSTOM 27: 3–97.

[B43] Guérin C (1980) Les rhinocéros (Mammalia, Perissodactyla) du Miocène terminal au Pléistocène supérieur en Europe occidentale; comparaison avec les espèces actuelles. Documents du Laboratoire de Géologie de la Faculté des Sciences de Lyon 79: 3–1183.

[B44] Hariyadi ARS Sajuthi D Astuti DA Alikodra HS Maheshwari H (2016) Analysis of nutrition quality and food digestibility in male Javan rhinoceros (*Rhinocerossondaicus*) in Ujung Kulon National Park. Pachyderm 57: 86–96. 10.69649/pachyderm.v57i.408

[B45] Harper F (1945) Extinct and vanishing mammals of the old world. New York, American Committee for International Wild Life protection, Special Publication no. 12: 1–850. 10.5962/bhl.title.19520

[B46] Haryono M Rahmat UM Daryan M Raharja AS Muhtarom A Firdaus AY Rohaeti A Subchiyatin I Nugraheni A Khairani KO Kartina (2015) Monitoring of Javan Rhino population in Ujung Kulon National Park. Pachyderm 56: 82–86. 10.69649/pachyderm.v56i.374

[B47] Hazarika BC Saikia PK (2010) A study on the behaviour of great Indian one-horned rhinoceros (*Rhinocerosunicornis*) in the Rajiv Gandhi National Park, Assam, India. NeBio 1(2): 62–74. 10.5402/2012/259695

[B48] Heißig K (1969) Die Rhinocerotidae (Mammalia) aus der oberoligozänen Spaltenfüllung von Gaimersheim bei Ingolstadt in Bayern und ihre phylogenetische Stellung. Verlag der Bayerischen Akademie der Wissenschaften; in Kommission bei der Beck‘schen CH Verlagsbuchhandlung, München: 1–133.

[B49] Heißig K (1972) Paläontologische und geologische Untersuchungen im Tertiär von Pakistan, 5. Rhinocerotidae (Mammalia.) aus den unteren und mittleren Siwalik-Schichten. Bayerische Akademie der Wissenschaften Mathematisch−Naturwissenschaftliche Klasse, Abhandlungen, München. Neue Folge 152: 1–112.

[B50] Heißig K (1973) Die Unterfamilien und Tribus der rezenten und fossilen Rhinocerotidae (Mammalia). Säugetierkundliche Mitteilungen 21: 25–30.

[B51] Heißig K (1981) Probleme bei der cladistischen Analyse einer Gruppe mit wenigen eindeutigen Apomorphien: Rhinocerotidae. Palaeontologisches Zeitschrift 55(1): 117–123. 10.1007/BF02986041

[B52] Heißig K (1989) 21 The Rhinocerotidae. In: Prothero DR Schoch RM (Eds) The Evolution of Perissodactyls.Oxford University Press, New York, 399–417.

[B53] Hernesniemi E Blomstedt K Fortelius M (2011) Multi-view stereo three-dimensional reconstruction of lower molars of Recent and Pleistocene rhinoceroses for mesowear analysis. Palaeontologia Electronica: 1–15.

[B54] Heude PM (1892) Études odontologiques. 1. Herbivores trizygodontes et dizygodontes. Mémoires concernant l’histoire naturelle de l’Empire Chinois 2: 65–84.

[B55] Hitchins PM (1986) Earlessness in the black rhinoceros–a warning. Pachyderm 7: 8–10. 10.69649/pachyderm.v7i1.634

[B56] Hoogerwerf A (1970) Udjung Kulon, the land of the last Javan rhinoceros. With local and general data on the most important faunal species and their preservation in Indonesia. Brill RJ, Leiden, 512 pp. 10.1163/9789004646698

[B57] Hooijer DA (1946) The evolution of the skeleton of *Rhinocerossondaicus* Desmarest. Proceedings of the Koninklijke Nederlandse Akademie van Wetenschappen, Amsterdam 49(6): 671–676.

[B58] Hooijer DA (1964) New records of mammals from the middle pleistocene of Sangiran, central Java. Zoologische Mededelingen 40(10): 73–88.

[B59] Hullot M Antoine P-O Ballatore M Merceron G (2019) Dental microwear textures and dietary preferences of extant rhinoceroses (Perissodactyla, Mammalia). Mammal Research 64: 397–409. 10.1007/s13364-019-00427-4

[B60] Jäger GF (1839) Über die fossilen Säugethiere welche in Württemberg in verschiedenen Formationen aufgefunden worden sind, nebst geognostischen Bemerkungen über diese Formationen. Verlag Carl Erhard, Stuttgart. Abtheilung 2: 1–214.

[B61] Khan AM (2009) Taxonomy and distribution of rhinoceroses from the Siwalik Hills of Pakistan. PhD Thesis, University of the Punjab, Lahore.

[B62] Kim HL Li T Kalsi N Nguyen HTT Shaw TA Ang KC Cheng KC Ratan A Peltier WR Samanta D Pratapneni M Schuster SC Horton BP (2023) Prehistoric human migration between Sundaland and South Asia was driven by sea-level rise. Commununications Biology 6: 1–10. 10.1038/s42003-023-04510-0 PMC9899273 36739308

[B63] Koenigswald GHR van (1935) Die fossilen Säugetierfaunen Javas. Proceedings van de Koninklijke Nederlandse Akademie van Wetenschappen 38: 88–98.

[B64] Laurie WA (1978) The ecology and behaviour of the greater one-horned rhinoceros. Ph.D. dissertation. University of Cambridge, Cambridge.

[B65] Laurie WA (1982) Behavioural ecology of the Greater one-horned rhinoceros (*Rhinocerosunicornis*). Journal of Zoology, London 196(3): 307–341. 10.1111/j.1469-7998.1982.tb03506.x

[B66] Laurie WA Lang RM Groves CP (1983) *Rhinocerosunicornis*. Mammalian Species 211, The American Society of Mammalogists: 1–6. 10.2307/3504002

[B67] Lesson RP (1836) Complement des œuvres de Buffon ou histoire naturelle des animaux rares découverts par les naturalistes et les voyageurs depuis la mort de Buffon, vol. 10: Oiseaux et Mammifères. Histoire naturelle générale et particulière des mammifères et oiseaux découverts depuis la mort de Buffon: Oiseaux et mammifères. Pourrat Frères et Roret, Paris, 414 pp.

[B68] Linnaeus C (1758) Systema naturae per regna tria naturae, secundum classes, ordines, genera, species, cum characteribus, differentiis, synonymis, locis Editio decima, reformata [edn 10]. Holmiae, Laurentii Salvii: i–iv, 1–824. 10.5962/bhl.title.542

[B69] Liu S Dalen L Gilbert T Rookmaaker LC and 35 others (2021) Ancient and modern genomes unravel the evolutionary history of the rhinoceros family. Cell 184: 4874–4885. 10.1016/j.cell.2021.07.032 34433011

[B70] Loch CW (1937) *Rhinocerossondaicus*: the Javan or Lesser one-horned rhinoceros and its geographical distribution. Journal of the Malayan Branch of the Royal Asiatic Society 15(2): 130–149.

[B71] Longuet M Zin-Maung-Maung-Thein Thaung-Htike Man-Thit-Nyein Masanaru (2023) New fossil remains of Rhinocerotidae (Perissodactyla) from the early Late Miocene Tebingan area, central Myanmar, Historical Biology: 1–14. 10.1080/08912963.2024.2408617

[B72] Longuet M Handa N Maung-Thein ZM Htike T Nyein MT Takai M (2024) Post-cranial remains of Rhinocerotidae from the Neogene of central Myanmar: morphological descriptions and comparisons with ratios. Historical Biology: 1–15. 10.1080/08912963.2024.2408617

[B73] Lydekker R (1907) The game animals of India, Burma and Tibet. Being a new and revised edition of “The great and small game of India, Burma and Tibet”. Rowland Ward, London: 1–409. 10.5962/bhl.title.16137

[B74] MacFadden BJ (1998) Tale of two rhinos: isotopic ecology, paleodiet, and niche differentiation of *Aphelops* and *Teleoceras* from the Florida Neogene. Paleobiology 24(2): 274–286. 10.1666/0094-8373(1998)024[0274:TOTRIE]2.3.CO;2

[B75] Margaryan A Sinding MS Liu S Vieira FG Chan YL Nathan S Moodley Y Bruford MW Gilbert MTP (2020) Recent mitochondrial lineage extinction in the critically endangered Javan rhinoceros. Zoological Journal of the Linnean Society 190(1): 372–383. 10.1093/zoolinnean/zlaa004

[B76] Matschiner M (2021) Museum specimens tell the history of rhinoceroses. Cell 184(19): 4841–4842. 10.1016/j.cell.2021.08.026 34499855

[B77] Matthew WD (1929) Critical observations upon Siwalik Mammals. Bulletin of the American Museum of Natural History, New York 56(7): 437–560.

[B78] Matthew WD (1931) Critical observations on the phylogeny of the rhinoceroses. University of California Publications, in Geological Science 20: 1–8.

[B79] McNeely JA Cronin EW (1972) Rhinos in Thailand. Oryx 11(6): 457–460. 10.1017/S0030605300010735

[B80] Medway L (1969) The wild mammals of Malaya and offshore islands, including Singapore. Kuala Lumpur, Oxford University Press: 1–127.

[B81] Morley RJ (2018) Assembly and division of the South and South-East Asian flora in relation to tectonics and climate change. Journal of Tropical Ecology 34(4): 209–234. 10.1017/S0266467418000202

[B82] Nardelli F (1988) The Rhinoceros: a Monograph. Basilisk Press, London, 133 pp.

[B83] Nardelli F (2013) The mega-folivorous mammals of the rainforest: feeding ecology in nature and in controlled environment: A contribution to their conservation. International Zoo News 60(5): 323–339.

[B84] Nardelli F (2016) Current status and conservation prospects for the Javan rhinoceros *Rhinocerossondaicus* Desmarest 1822. International Zoo News 63(3): 180–202.

[B85] Nardelli F Robovský J (2023) New data on the ecology and conservation of the Javan rhinoceros *Rhinocerossondaicus* Desmarest, 1822 (Perissodactyla, Rhinocerotidae). Gazella (Praha) 49(2022): 183–205.

[B86] Neese HC (1975) Survival of the Javan rhinoceros in Laos. Report: 1–27.

[B87] Owen R (1870) On fossil remains of mammals found in China. Quarterly Journal of the Geological Society London 26: 417–434. 10.1144/GSL.JGS.1870.026.01-02.40

[B88] Owen-Smith RN (1988) Megaherbivores: The influence of very large body size on ecology. Cambridge University Press, Cambridge, 369 pp. 10.1017/CBO9780511565441

[B89] Pandolfi L (2015) Sistematica e filogenesi dei Rhinocerotini (Mammalia, Rhinocerotidae). Dissertation presented to the University of Rome, Italy.

[B90] Pandolfi L Maiorino L (2016) Reassessment of the largest Pleistocene rhinocerotine *Rhinocerosplatyrhinus* (Mammalia, Rhinocerotidae) from the Upper Siwaliks (Siwalik Hills, India). Journal of Vertebrate Paleontology 36(2): 1–12. 10.1080/02724634.2015.1071266

[B91] Pandolfi L Bartolini-Lucenti S Cirilli O Bukhsianidze M Lordkipanidze D Rook L (2021) Paleoecology, biochronology, and paleobiogeography of Eurasian Rhinocerotidae during the Early Pleistocene: The contribution of the fossil material from Dmanisi (Georgia, Southern Caucasus). Journal of Human Evolution 156: 1–13. 10.1016/j.jhevol.2021.103013 34030060

[B92] Peacock EH (1933) A game book for Burma and adjoining territories. H&F, Witherby G, London, 292 pp.

[B93] Piras P Maiorino L Raia P Marcolini F Salvi D Vignoli L Kotsakis T (2010) Functional and phylogenetic constraints in Rhinocerotinae craniodental morphology. Evolutionary Ecology Research 12: 897–928.

[B94] Poole CM Duckworth JW (2005) A documented 20^th^ century record of Javan Rhinoceros*Rhinocerossondaicus* from Cambodia. Mammalia 69(3–4): 443–444. 10.1515/mamm.2005.039

[B95] Reynolds EAP (1954) Burma rhino. Burmese Forester 4(2): 104–108.

[B96] Rookmaaker LC (1977) The rhinoceros of Borneo: a 19^th^ century puzzle. Journal of the Malayan Branch of the Royal Asiatic Society 50(1): 52–62.

[B97] Rookmaaker LC (1980) The distribution of the rhinoceros in Eastern India, Bangladesh, China and the Indo-Chinese region. Zoologische Anzeiger 205(3/4): 253–268.

[B98] Rookmaaker LC (1997) Records of the Sundarbans rhinoceros (*Rhinocerossondaicusinermis*) in India and Bangladesh. Pachyderm 24: 37–45. 10.69649/pachyderm.v24i1.903

[B99] Rookmaaker LC (2000) Records of the rhinoceros in Pakistan and Afghanistan. Pakistan Journal of Zoology 32(1): 65–74.

[B100] Rookmaaker LC (2002) Historical records of the Javan rhinoceros in North-East India. Newsletter of the Rhino Foundation of Nature in North-East India 4: 11–12.

[B101] Rookmaaker LC (2006) Distribution and extinction of the rhinoceros in China: review of recent Chinese publications. Pachyderm 102: 102–106. 10.69649/pachyderm.v40i1.1267

[B102] Rookmaaker LC (2016) On the alleged presence of the two-horned Sumatran rhinoceros and one-horned Javan rhinoceros in the Himalayan Kingdom of Bhutan. Pachyderm 57: 116–117. 10.69649/pachyderm.v57i.399

[B103] Rookmaaker LC Carpentier H (2007) Early references to the rhinoceros on the Chinese island of Hainan. Journal of the Royal Asiatic Society Hong Kong Branch 45(2005): 235–236.

[B104] Schaller GB Dang NX Thuy LD Son VT (1990) Javan rhinoceros in Vietnam. Oryx 24(2): 77–80. 10.1017/S0030605300034712

[B105] Schellhorn R (2018) A potential link between lateral semicircular canal orientation, head posture, and dietary habits in extant rhinos (Perissodactyla, Rhinocerotidae). Journal of Morphology 279: 50–61. 10.1002/jmor.20753 28948643

[B106] Setiawan R Gerber B Ujang MR Daryang D Firdaus AY Haryono M Khairani KO Kurniawan Y Long B Lyet A Muhiban M Mahmud R Muhtarom A Purastuti E Ramono W Subrata D Sunarto S (2017) Preventing global extinction of the Javan Rhino: Tsunami risk and future conservation direction. Conservation Letters 11(1): 1–9. 10.1111/conl.12366

[B107] Siddiq MKh Akhtar M Khan MA Ghaffar A Sarwar Kh Khan AM (2016) New fossils of rhinoceros (Rhinocerotidae) from the Soan Formation (Plio-Pleistocene) of Northern Pakistan. Pakistan Journal of Zoology 48(3): 657–664.

[B108] Singh S Parkash B Awasthi AK Singh T (2012) Palaeoprecipitation record using O-isotope studies of the Himalayan Foreland Basin sediments, NW India. Palaeogeography, Palaeoclimatology, Palaeoecology 331–332: 39–49. 10.1016/j.palaeo.2012.02.031

[B109] Sody HJV (1959) Das Javanische Nashorn, *Rhinocerossondaicus*, historisch und biologisch. Zeitschrift fur Saugetierkunde 24(3/4): 109–240.

[B110] Strien NJ van (1985) The Sumatran rhinoceros in the Gunung Leuser National Park, its distribution, ecology and conservation. Doorn, Van Strien: 1–207.

[B111] Suraprasit K Jaeger JJ Chaimanee Y Chavasseau O Yamee C Tian P Panha S (2016) The Middle Pleistocene vertebrate fauna from Khok Sung (Nakhon Ratchasima, Thailand): biochronological and paleobiogeographical implications. ZooKeys 613: 1–157. 10.3897/zookeys.613.8309 PMC5027644 27667928

[B112] Tougard C Delefosse T Hoenni C Montgelard C (2001) Phylogenetic relationships of the five extant rhinoceros species (Rhinocerotidae, Perissodactyla) based on mitochondrial cyctochrome b and 12s rRNA genes. Molecular Phylogenetics and Evolution 19(1): 34–44. 10.1006/mpev.2000.0903 11286489

[B113] Toula F (1902) Das Nashorn von Hundsheim *Rhinoceros* (*Ceratorhinus* Osborn) *hundsheimensis* nov. form. mit Ausführungen über die Verhältnisse von elf Schädeln von Rhinoceros (Ceratorhinus) sumatrensis. Abhandlungen der Kaiserlich-Königlichen Geologischen Reichsanstalt 19: 1–92.

[B114] Willerslev E Gilbert M Binladen J Ho S Campos P Ratan A Tomsho L Fonseca R da Sher A Kuznetsova T Nowak-Kemp M Roth T Miller W Schuster S (2009) Analysis of complete mitochondrial genomes from extinct and extant rhinoceroses reveals lack of phylogenetic resolution. BMC Evolutionary Biology 9: 1–30. 10.1186/1471-2148-9-95 19432984 PMC2694787

[B115] Wilson SG (2021) Factors shaping the conservation of the critically endangered Javan Rhinoceros–*Rhinocerossondaicus*. Ph.D. thesis, School of Biological Sciences, University of Queensland, Brisbane.

[B116] Yan Y Wang Y Zhu M Zhang Y Qin D Jin C (2023) New rhino remains from Middle to Late Pleistocene of Chongzuo, Guangxi with discussion on Quaternary Rhinoceros evolution in Southern China. Quaternary Sciences 43(3): 777–792.

[B117] Zeuner FE (1934) Die Beziehungen zwischen Schädelform und Lebensweise bei den rezenten und fossilen Nashornern. Berichte der Naturforschenden Gesellschaft zu Freiburg im Breisgau 34: 21–80.

[B118] Zin-Maung-Maung-Thein Thaung-Htike Tsubamoto T Takai M Egi N Maung-Maung (2006) Early Pleistocene Javan rhinoceros from the Irawaddy Formation, Myanmar. Asian Paleoprimatology, Primate Research Institute, Kioto University 4: 197–204.

[B119] Zin-Maung-Maung-Thein Takai M Tsubamoto T Egi N Thaung-Htike Nishimura T Maung-Maung Zaw-Win (2010) A review of fossil rhinoceroses from the Neogene of Myanmar with description of new specimens from the Irrawaddy Sediments. Journal of Asian Earth Sciences 37(2): 154–165. 10.1016/j.jseaes.2009.08.009

[B120] Zschokke S (2016) Genetic structure of the wild populations of the Indian rhinoceros (*Rhinocerosunicornis*). Indian Journal of History of Science 51(2.2): 380–389. 10.16943/ijhs/2016/v51i2.2/48451

[B121] Zschokke S Armbruster GFJ Ursenbacher S Baur B (2011) Genetic differences between the two remaining wild populations of the endangered Indian rhinoceros (*Rhinocerosunicornis*). Biological Conservation 144: 2702–2709. 10.1016/j.biocon.2011.07.031

